# Novel Nanocomposite PLA Films with Lignin/Zinc Oxide Hybrids: Design, Characterization, Interaction with Mesenchymal Stem Cells

**DOI:** 10.3390/nano10112176

**Published:** 2020-10-31

**Authors:** Francesca Luzi, Ilaria Tortorella, Alessandro Di Michele, Franco Dominici, Chiara Argentati, Francesco Morena, Luigi Torre, Debora Puglia, Sabata Martino

**Affiliations:** 1 Department of Civil and Environmental Engineering, Materials Engineering Center, UdR INSTM, University of Perugia, Strada di Pentima 4, 05100 Terni, Italy; francesca.luzi@unipg.it (F.L.); franco.dominici@unipg.it (F.D.); luigi.torre@unipg.it (L.T.); 2 Department of Chemistry, Biology and Biotechnologies, University of Perugia, 06123 Perugia, Italy; tortorella.i@hotmail.it (I.T.); chiara.argentati89@gmail.com (C.A.); francesco.morena@unipg.it (F.M.); 3 Department of Physics and Geology, University of Perugia, Via Pascoli, 1, 06123 Perugia, Italy; alessandro.dimichele@collaboratori.unipg.it; 4 CEMIN, Center of Excellence on Nanostructured Innovative Materials, Via del Giochetto, 06123 Perugia, Italy

**Keywords:** Poly(lactic acid), lignin nanoparticles, zinc oxide nanoparticles, bone-marrow mesenchymal stem cells (hBM-MSCs), human adult adipose stem cells (hASCs), biomedical application, food packaging application

## Abstract

Herein we present the production of novel nanocomposite films consisting of polylactic acid (PLA) polymer and the inclusion of nanoparticles of lignin (LNP), ZnO and hybrid ZnO@LNP (ZnO, 3.5% wt, ICP), characterized by similar regular shapes and different diameter distribution (30–70 nm and 100–150 nm, respectively). The obtained set of binary, ternary and quaternary systems were similar in surface wettability and morphology but different in the tensile performance: while the presence of LNP and ZnO in PLA caused a reduction of elastic modulus, stress and deformation at break, the inclusion of ZnO@LNP increased the stiffness and tensile strength (σ_b_ = 65.9 MPa and E_Young_ = 3030 MPa) with respect to neat PLA (σ_b_ = 37.4 MPa and E_Young_ = 2280 MPa). Neat and nanocomposite PLA-derived films were suitable for adult human bone marrow-mesenchymal stem cells and adipose stem cell cultures, as showed by their viability and behavior comparable to control conditions. Both stem cell types adhered to the films’ surface by vinculin focal adhesion spots and responded to the films’ mechanical properties by orchestrating the F-actin–filamin A interaction. Collectively, our results support the biomedical application of neat- and nanocomposite-PLA films and, based on the absence of toxicity in seeded stem cells, provide a proof of principle of their safety for food packaging purposes.

## 1. Introduction

Polylactic acid (PLA) is considered a high potential material, owing to its excellent mechanical properties, biodegradability, biocompatibility, high transparency and commercial availability. Nonetheless, some drawbacks such as limited protection against various physical, chemical, biological and environmental conditions (oxygen, moisture, light, microorganisms, and physical stress) somewhat limit its widespread access to specific industrial applications, for instance in packaging and biomedical sectors. Recently, lignin (the most abundant natural material after cellulose) has also drawn much attention due to its multi-functionalities, including antioxidant ultraviolet (UV)-absorbing and antimicrobial properties, which can be used in nanocomposites for food packaging, drug delivery and gene delivery vehicles for biomedical application [1,2]. In addition, its use in PLA is considered particularly attractive, being a biobased and biodegradable material. Quite recently, the introduction of lignin at the nanoscale has also been proved to be effective in enhancing the overall mechanical, thermal and antioxidant behavior of PLA matrix. Moreover, there are already some studies that offer information about the effect of the inclusion of lignin nanoparticles (LNP) into PLA by different methods, such as melt blending [3].

In parallel, additives based on inorganic metal oxide nanoparticles (TiO_2_, ZnO, Fe_2_O) [4,5] have been considered for incorporation into different supporting matrices, with the aim of enhancing the antioxidant and antibacterial activity of polymeric films [6]. Zinc oxide (ZnO) nanoparticles have the advantage, over other nanoparticles, of having low cost, easy availability, antibacterial effects, and intensive ultraviolet absorption. Moreover, nano-ZnO has been approved by the Food and Drug Administration (FDA) as a safe material [7]. Hence, as an important functional inorganic material, nano-ZnO is increasingly used in research and health-related applications. Even though the exact mechanism of the antibacterial action of ZnO nanoparticles is still not entirely clarified, several hypotheses have been made: a) inhibition of bacterial cell growth due to the generation of hydrogen peroxide (H_2_O_2_) at the surface of ZnO particles [8], b) zinc ion release [9], c) mechanical damage of the cell wall through adhesion on the cell membrane, penetration through the membrane cell wall, and cellular internalization of nanoparticles [10,11]. The morphology of ZnO particles represents an important factor: in particular, the ZnO powder with the largest specific surface area and the smallest particle size has the highest antibacterial activity [12]. Based on literature information we aimed to consider, in our experimental work, a spherical geometry of synthesized ZnO nanoparticles. Limiting the analysis to the zinc oxide nanoparticles, their combination with PLA has been already extensively considered [13,14], confirming the important role of this nanofiller in tuning structural and functional properties of produced films.

Furthermore, lignin has been described as a three-dimensional network natural polymer which is beneficial for forming a neat uniform composite structure with inorganic nanoparticles [15], thus organic–inorganic hybrid materials have elicited much attention in recent years. The preparation of lignin/inorganic nanocomposites, potentially combining the advantages of all components to access complementary properties and synergetic effects provides a new approach for the high-valued application of industrial lignin. There are few reports in the literature regarding the preparation of this type of system, since emphasis is put primarily on the possibility of using lignin as a precursor for metal oxide synthesis to be further incorporated in polymeric matrices [16,17], while limited works are available dealing with the incorporation of lignin nanoparticles, in the presence of other inorganic metal oxide nanoparticles, with the main aim of synergically combining the two nanofillers to enhance ultraviolet (UV), antioxidant and antibacterial protection of biodegradable polymer matrix [18,19]. It has been proved that the concurrent addition of lignin and ZnO to polymeric matrices, as in the case of polyurethane, can significantly change their properties since lignin/ZnO hybrid nanocomposites may have unexpected excellent UV optical properties and the toughness of lignin and stiffness of ZnO may combine. Furthermore, both nanoparticles were demonstrated to be non-toxic to human cells by several studies. This aspect allowed for their use as antibacterial agents, toxic to bacteria and harmless to human cells [20,21].

Nevertheless, to the best of our knowledge, the preparation of lignin/ZnO hybrid PLA nanocomposites has not been reported so far. Given this, here we considered the combination of LNP and ZnO with their hybrid material (ZnO@LNP), taking as main aim the verification of their possible synergic effect on the overall morphological, thermal and mechanical performance of the films produced. Neat PLA and PLA nanocomposites containing LNP, ZnO or ZnO@LNP, alone or in combination to generate binary, ternary and quaternary systems, were produced. Moreover, an extensive study on the biocompatibility of the different materials has been conducted, aiming to confirm their promising use in food and biomedical applications, where biocompatibility is the basic requirement [22,23,24]. In this regard, we evaluated the effect of the interaction of neat and PLA polymeric nanocomposite films with adult human bone marrow-mesenchymal stem cells (hBM-MSCs) and adult human adipose stem cells (hASCs). We have selected these stem cell types based on our previous studies highlighting the capability of both stem cell types to adhere, proliferate and adapt their morphology to the surface characteristics of polymeric films [25,26,27,28,29]. Additionally, both stem cell types can be cultured in vitro with a relatively simple and standardized protocol [25,26,27,28,29] that allows monitoring the stem cell behavior during the period of culture. Here, we cultured both hBM-MSCs and hASCs on films of neat and PLA nanocomposites and analyzed their effect on stem cell proliferation, adhesion and shape.

## 2. Materials and Methods

### 2.1. Materials

Alkali lignin, hydrochloric acid (HCl, 35%), ethylene glycol (C_2_H_6_O_2_, 99.8%), zinc acetate dihydrate ((CH_3_CO_2_)_2_Zn × 2H_2_O), zinc nitrate hexahydrate (Zn(NO_3_)_2_ × 6H_2_O), sodium bicarbonate (NaHCO_3_) and hexamethylenetetramine (C_6_H_12_N_4_) were supplied by Sigma-Aldrich, St. Louis, MO, USA. Poly(lactic acid) (PLA 3251D), with a specific gravity of 1.24 g/cm^3^, a relative viscosity of ca. 2.5, and melt flow index (MFI) of 35 g/10 min (190 °C, 2.16 kg) was supplied by Nature Works LLC, MN, USA.

### 2.2. Preparation of Lignin and Zinc Oxide Nanoparticles and Their Characterization

*LNP:* LNP suspension was obtained from alkali lignin by hydrochloric acid treatment based on the procedure reported in previous papers [30,31,32,33]. 4% (*m*/*v*) of alkali lignin in ethylene glycol was stirred for 2 h at 35 °C. Afterward, hydrochloric acid (8 mL, 0.25 M) was mildly added to the solution at a rate of 3−4 drops/min, then the suspension was stirred again for another 2 h. The product was filtered to eliminate soluble impurities from lignin. The solution was then dialyzed against deionized water up to neutrality to obtain the LNP suspension.

*ZnO:* For the synthesis, 100 mL of NaHCO_3_ 0.15 M were dropped, under ultrasound irradiation for 1 h at 50% of amplitude at 70 °C and under Argon flow, to a 200 mL of a zinc acetate 0.1 M. The ZnO nanoparticles were synthesized by using a method proposed in the literature with some modifications [34]. The precipitate was centrifuged, washed and calcined at 350 °C for 1 h.

*ZnO@LNP:* For the synthesis, 250 mL of a mixture of lignin (2.76 g), zinc nitrate (0.57 g) and hexamethylenetetramine (0.42 g) was treated with high power ultrasound for 45 min at 50% of amplitude at 90 °C and under argon flow. After the synthesis, the precipitate was centrifuged, washed and dried. The high-power ultrasound irradiation was performed by using an Ultrasonic processor VCX750 (Sonics & Materials, Inc., CT, USA), 20 kHz, with a diameter tip of 13 mm.

*FESEM*: Drops of aqueous suspensions of LNP, ZnO and ZnO@LNP were deposited on silicon substrates, air-dried for 24 h, chromium coated by using an ion sputter coater, and observed by using field-emission scanning electron microscopy (FESEM Supra 25, Zeiss, Oberkochen, Germany) and a field-emission gun operated at 5 kV.

Elemental composition and chemical mapping were determined by using a Quantax Energy Dispersive Spectroscopy (EDS) (Bruker, MA, USA) operating at 15 kV.

Thermogravimetric measurements (TGA) of nanoparticle powders were performed under nitrogen flow (250 mL min^−1^) by using a Seiko Exstar 6300 (Seiko Instruments Inc., Chiba, Japan), applying a heating scan from 30 to 900 °C at 10 °C min^−1^.

*ICP:* ZnO analysis was performed, by using a Varian 700-ES series inductively coupled plasma-optical emission spectrometer (ICP-OES) (Agilent Technologies, Santa Clara, CA, USA) on solutions prepared by dissolving the ZnO@LNP with 2 mL of HNO_3_ 65% wt and 1 mL of H_2_O_2_ 35% *v*/*v* and properly diluted.

### 2.3. Preparation of Polylactic Acid (PLA) and PLA Nanocomposite-Based Films

PLA pellets were dried overnight at 45 °C before extrusion to prevent polymer hydrolysis during the processing phase [3,35]. Different formulations with LNP, ZnO and ZnO@LNP nanoparticles were realized by using a twin-screw microextruder (DSM Explore 5 and 15 CC Micro Compounder, Xplore instruments BV, Sittard, The Netherlands).

Screw speed of 90 rpm and total mixing time of 3 min were used to optimize the final properties of the material, while a temperature profile from 180 °C to a maximum mixing temperature of 190 °C was chosen. LNP (2 wt.%), ZnO (0.5 wt.%) and ZnO@LNP (0.5 wt.%) were added after 2 min. The thickness of the developed films was determined at five random positions using a 293 MDC-Lite Digimatic Micrometer (Mitutoyo, Kawasaki, Japan), obtaining average thickness values of around 40 µm. We have generated a set of binary, ternary and quaternary films by setting PLA as the unfilled reference film. In Table 1, chemical formulation, acronyms and percentage of LNP, ZnO and ZnO@LNP that were specifically added to the basic PLA are reported. According to the different compositions, we obtained PLA(P)-binary (P_2L; P_05Z; P_05Z@L), PLA-ternary (P_2L_05Z; P_2L _05Z@L; P_05Z _05Z@L) and PLA quaternary (P_2L_05Z_05Z@L) films.

Film strips of a few meters in length, about 50 mm wide and about 40 µm thick, were obtained.

### 2.4. Characterization of PLA-Based Films

The microstructure (surface and fractured cross-section) of PLA and PLA nanocomposite films was investigated by field-emission scanning electron microscopy (FESEM, Supra 25, Zeiss, Oberkochen, Germany). The surfaces were gold sputtered in order to provide electric conductivity and the samples were observed using an accelerating voltage of 2.5 kV.

Tensile tests of neat PLA and PLA films were performed to determine the effect of LNP, ZnO and ZnO@LNP addition on the mechanical response of the PLA matrix. The test was performed on rectangular samples according to UNI ISO 527. Ultimate tensile strength (σ_max_), elongation at break (ε_b_), and Young’s modulus (E_Young_) were calculated from the resulting stress–strain curves. The measurements were taken at room temperature and at least five samples for each formulation were tested.

Surface wettability of the PLA films was studied through static water contact angle measurements with a standard goniometer (FTA2000, First Ten Angstroms, Inc., Newark, CA, USA) equipped with a camera and Drop Shape Analysis SW21; FTA32 2.0 software (First Ten Angstroms, Inc., Portsmouth, UK; accessed on 20 September 2011) was used to test the water contact angle at room temperature. The contact angle was determined by randomly putting 5 drops of distilled water (2 µL) with a syringe onto the film surfaces and, after 1 s, the average values of five measurements for each drop were used.

### 2.5. Protein Adsorption

Protein adsorption assays were carried out following our previously published protocol [36]. All PLA films (0.5 cm^2^, square shaped) were incubated with 2 mg/mL of bovine serum albumin (BSA, Sigma-Aldrich, St. Louis, MO, USA) and 10% of fetal bovine serum (FBS, Euroclone S.p.A, Pero (MI), Italy) for 30 min and 24 h at 37 °C. After three washing steps with deionized water (dH_2_O), total adsorbed protein content was evaluated using the Bradford method [37]. Absorbance (595 nm) was measured using a microtiter plate reader (enzyme-linked immunosorbent assay (ELISA) reader, GDV-DV990BV6, Roma, Italy). Each sample was analyzed in three independent experiments. Data are reported as mean ± standard deviation.

### 2.6. Isolation and Culture of Human Adult Mesenchymal Stem Cells

#### 2.6.1. Adipose Stem Cells

Human adipose stem cells (hASCs), were isolated as described by our group [25,27,28,29]. hASCs were isolated from waste samples from surgery to which adult donor subjects were subjected. The procedure was occasional and is not part of a specific project. All procedures were done with the consent of donors and in accordance with the Declaration of Helsinki. Briefly, after extensively washing in phosphate-buffered saline (PBS) containing 5% penicillin/streptomycin (Euroclone S.p.A, Pero (MI), Italy), fragments were incubated 40 min at 37 °C, 5% carbon dioxide (CO_2_), with 0.075% collagenase from clostridium (Sigma Aldrich, St. Louis, MO, USA) for tissue digestion prepared in PBS containing 0.5% of BSA. The digested fragments were centrifuged at 300× *g* for 5 min and the pellet obtained was washed with PBS 2% penicillin/streptomycin, and centrifuged at 300× *g* for 5 min. Finally, the cell pellet was re-suspended in growth medium, RPMI-1640 (Euroclone S.p.A, Pero (MI), Italy) supplemented with 10% of heat-inactivated fetal bovine serum (FBS, Euroclone S.p.A, Pero (MI), Italy), 1% l-glutamine (Euroclone S.p.A, Pero (MI), Italy), 1% penicillin/streptomycin, plated in tissue culture flasks (TCP) and then incubated at 37 °C, 5% CO_2_. hASCs started to grow as adherent fibroblast-like cells and every three days the medium was changed.

#### 2.6.2. Bone-Marrow Mesenchymal Stem Cells

Human bone marrow-mesenchymal stem cells (hBM-MSCs) were isolated as described by our group [28,36]. hBM-MSCs were isolated from waste samples (washouts of the medullary cavities of patients’ femurs) from surgery to which adult donor subjects were subjected. The procedure was occasional and is not part of a specific project. All procedures were done with the consent of donors and in accordance with the Declaration of Helsinki. hBM-MSCs were isolated from the mononuclear cells of the density gradient (Lympholyte; Cedarlane Laboratories Limited, Hornby, ON, Canada) component of the bone marrow, by seeding in culture flasks in growth culture medium: Dulbecco’s Modified Eagle Medium (DMEM) high glucose (Euroclone S.p.A, Pero (MI), Italy) medium containing FBS 10%, 2 mM L-glutamine, and 1% penicillin–streptomycin (Euroclone S.p.A, Pero (MI), Italy) in a humidified atmosphere and 5% CO_2_ at 37 °C. Non-adherent cells were removed after 5 to 7 days, and the fresh medium was added to the flasks. After 15 days, a fibroblast-like colony started to grow. Isolated were seeded in TCP culture flasks and kept in culture using containing 10% FBS, 1% 2 mM L-glutamine, 1% penicillin/streptomycin (Euroclone S.p.A, Pero (MI), Italy) in humidified atmosphere at 37 °C, 5% CO_2_. Medium change occurred every three days.

#### 2.6.3. Phenotypical Characterization of Mesenchymal Stem Cells

To assess mesenchymal phenotype, hASCs and hBM-MSCs were analyzed through flow cytometry FACScan (BD Biosciences) as previously reported [27] using the mesenchymal stem cell marker CD45, CD73, CD90 and CD105 (all from BD Biosciences, San Jose, CA, USA). Data analyses were performed with the FlowJo software (Tree Star, Ashland, OR, USA version 10.0.1, accessed on 2012).

### 2.7. Culture of Stem Cells on PLA Films

Film preparation: 1 cm^2^ squares of each PLA film type were sterilized by 30 s immersion in 70% ethanol, rinsed with sterile PBS and placed in multi-well plates.

Once dried, stem cells suspension was seeded drop wise on sterilized films. After 45 min the growth culture medium was gently added to each film. Stem cells on PLA films were incubated under canonical culture conditions in humidified atmosphere at 37 °C, 5% CO_2_. Medium was changed every three days and cell cultures were analyzed for assessing cell viability and morphology.

#### 2.7.1. Cell Viability Assay

Viability of hBM-MSCs and hASCs on PLA films was evaluated by using the MTT (3-(4,5-dimethylthiazol-2-yl)-2,5-diphenyltetrazolium bromide) assay (Sigma-Aldrich) according to the manufacturer’s recommendation. 1.5 × 10^3^ cells were seeded on 0.5 cm^2^ PLA films squares (placed in 48-well placed) and TCP as internal control. The interference effects of each polymer film snippet squares on MTT assay, were also considered without cells. The absorbance of the samples was measured at 3, 7 and 14 days in culture, using a microtiter plate reader (ELISA reader, GDV-DV990BV6, Roma, Italy) at 589 nm with a reference wavelength at 650 nm. Every experiment was performed in triplicate.

Stem cell proliferation was calculated by using the MTT absorbance regression curve generated by measuring the optical density (OD) of the assay performed in a serial number of both stem cells types.

#### 2.7.2. Immunofluorescences

Stem cells on PLA films and glass coverslips (GC) as internal control were rinsed twice with PBS, fixed in 4% paraformaldehyde for 20 min and, after PBS rinsing, permeabilized (PBS + 3% FBS + 0,5% Triton X-100) and blocked (PBS + 3% FBS + 0.05% Triton X-100) for 1 h at room temperature (RT). Samples were incubated with phalloidin (Alexa-fluor-488 phalloidin, Invitrogen, Grand Island, NY, USA) for 20 min at RT to achieve F-Actin staining, or overnight at 4 °C with primary human antibody anti-Filamin (Santa Cruz Biotechnology, Inc., Dallas, TX, USA) or anti-Vinculin (Abcam, Cambridge, UK), followed by incubation with secondary antibody conjugated with Alexa-Fluor-594 (Invitrogen) for 1h at RT. After washing with PBS, samples were mounted and nuclei were stained with Vectashield Antifade Mounting Medium with 4′,6-diamidino-2-phenylindole (DAPI) (Vector Laboratories Inc., Burlingame, CA, USA). Image acquisition was performed by using a fluorescence microscope (Eclipse-TE2000-S, Nikon, Tokyo, Japan) equipped with the F-ViewII FireWire camera (cell^f^ Soft Imaging System, Olympus, Germany, version 2.5, Accessed in 2006). Interference of PLA films without cells was also evaluated.

### 2.8. Cyto-Morphometric Measurements

The cell morphometric measurements were performed as previously reported [26] by using Fiji (Fiji Life-Line version 2.0.0, 2018) [38]. Cell shape index (CSI) and nuclear shape index (NSI) were determined on fluorescent-stained (Phalloidin-Alexa-fluor-488, DAPI) digital images of hASC and hBM-MSCs on each PLA film, acquired as previously described [26].

### 2.9. Statistical Analysis

Experimental data were reported as mean ± standard deviation (SD), median and range of intervals (boxplot). Post-hoc comparison test was carried out using the one-way analysis of variance (ANOVA) and Dunn’s multiple comparison test (GraphPad Software, Inc., La Jolla, CA, USA, version 4.0, Accessed in 2008). The *p* < 0.05 was considered statistically significant.

## 3. Results

### 3.1. Characterization of Lignin Nanoparticles (LNP), ZnO and ZnO@LNP Nanoparticles

**Figure 1:**
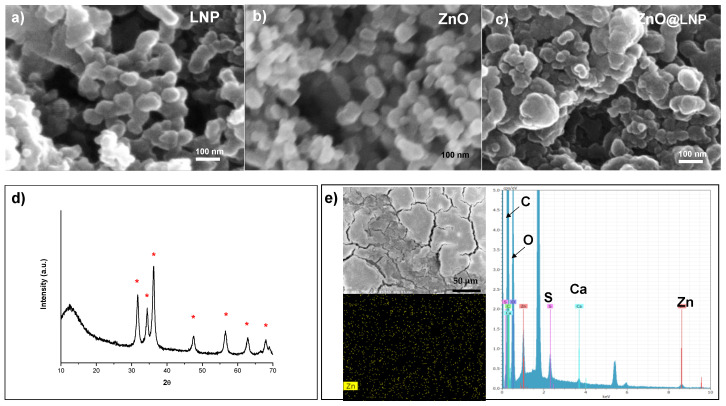
(**a**) Field-emission scanning electron microscopy (FESEM) images of lignin nanoparticles (LNP), (**b**) ZnO and (**c**) ZnO@LNP nanoparticles. X-ray diffraction (XRD) patterns (**d**) and Energy Dispersive X-Ray (EDX) analysis (**e**) of ZnO@LNP.

Firstly, we produced and characterized the different synthetized nanoparticles. As shown in Figure 1a, LNP nanoparticles have a regular shape with average diameters mainly ranging from 30 to 65 nanometers [31,32,33]. A slight aggregation of LNP is noted (Figure 1a), this phenomenon, essentially related to the pH of LNP solution (around 6.5), was already observed in [31]. Similar morphology was observed for ZnO nanoparticles. The oxide nanostructures appeared individualized with a regular shape and with an average diameter of 50–70 nm. FESEM image of ZnO shows nanostructures with higher homogeneous dimension distribution with respect to LNP (Figure 1b). The morphological properties of hybrid materials are influenced by the composition of individual components. The ICP analysis showed that the percentage of ZnO in the ZnO@LNP samples was 3.5% wt. The ZnO@LNP nanostructures also obtained in this case were individualized but they showed a higher diameter distribution (100–150 nm) (Figure 1c). This effect is due to the growth of ZnO nanostructures on the external surface of LNP, as demonstrated by the diffraction peaks of ZnO in the X-ray diffraction (XRD) spectrum (Figure 1d) and the homogeneous deposition of ZnO on lignin surface [39,40,41] (Figure 1e).

**Figure 2:**
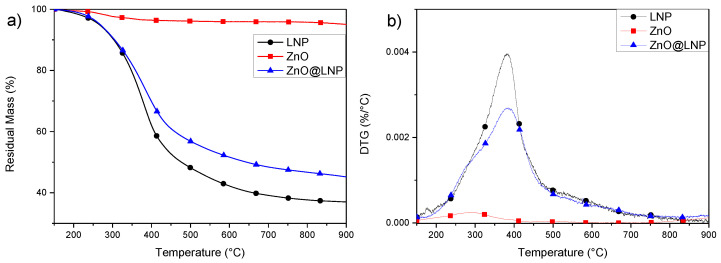
Residual mass (**a**) and Derivative mass loss (DTG) curves (**b**) of LNP, ZnO and ZnO@LNP nanoparticles.

Assessment of thermal-degradative stability was carried out, with the aim of studying the effect of the nanofillers on the characteristics of polymeric systems. Thermogravimetry (TG) and Derivative thermogravimetry (DTG) curves of LNP, ZnO and ZnO@LNP nanofillers are reported in Figure 2a,b. It was observed that thermal decomposition of LNP started at low temperature, at around 200 °C, and achieved the maximum degradation rate at the peak temperature (T_max_) of 382 °C [31,42]. The final weight loss was found at 900 °C equal to 31.5%. ZnO nanoparticles showed a maximum degradation peak centered at 290 °C, this degradation counts for only 3.5% in weight loss. This behavior highlights that this material is stable during the thermal event that occurs during heating in the extruder. The thermo-degradative study of the ZnO@LNP showed that this nanofiller started to degrade at 200 °C, with the presence of a shoulder centered at 290 °C related to the presence of ZnO, while the maximum peak is centered at around 382 °C, in parallel with a mass loss equal to 26.5%. However, the maximum degradation peak in LNP and hybrid nanofiller (ZnO@LNP) was centered at the same temperature. This behavior underlines that no variation in terms of degradation temperature with respect to LNP was registered by combining ZnO and LNP. A similar trend was observed also in literature, where thermal stability of ZnO–lignin and MgO–lignin hybrid systems has been studied [39,40,41,43].

### 3.2. Film Characterizations

**Figure 3:**
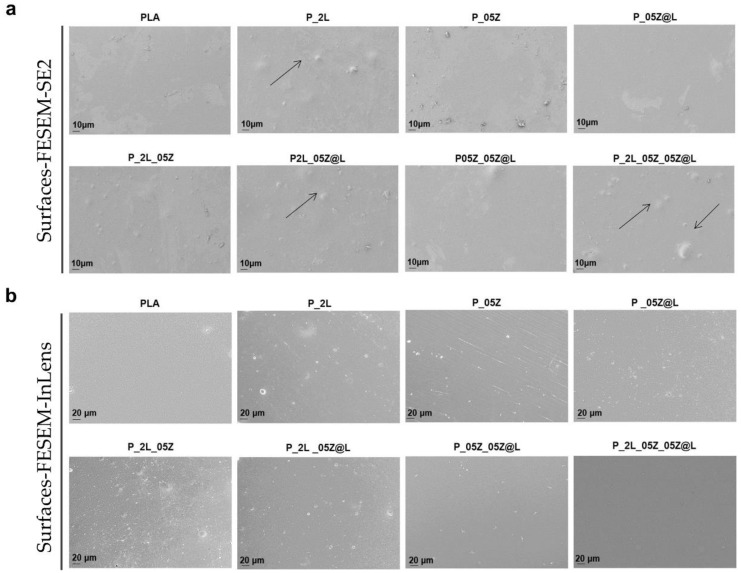
Microstructure surface investigation of PLA systems by using (**a**) FESEM and SE2 detector and (**b**) FESEM and InLens detector (arrows indicates the presence of nanoparticles on surface of the films).

Figure 3 shows the microstructure surfaces of different PLA films observed by FESEM. The morphological study of external surfaces was carried out by using two different detectors to better analyze the external surface of polymeric systems. These investigations permitted us to determine the effect of LNP, ZnO and ZnO@LNP nanofillers on the overall surface morphology of the reference neat PLA. The detector SE2 gives the possibility to analyze the roughness of the different surfaces, while the detector InLens permits us to observe the nanofiller distributions well. The surface of PLA film observed with the detector SE2 appears homogenous, without any defect [44]. A slight increase of roughness was observed by adding LNP in PLA films (see also the arrows) [45]. The presence and combination of ZnO and ZnO@LNP nanoparticles in binary based films (P_05Z and P_05Z@L) keeps unchanged the surface of PLA. On the surface of nanocomposites, the presence of white spots related to the presence and combination of nanoparticles is visible. In general, LNP, ZnO and ZnO@LNP appear homogenously dispersed (see images for detector SE2 and InLens). In conclusion, the presence of different nanofillers does not compromise the integrity of PLA-based formulations.

**Figure 4:**
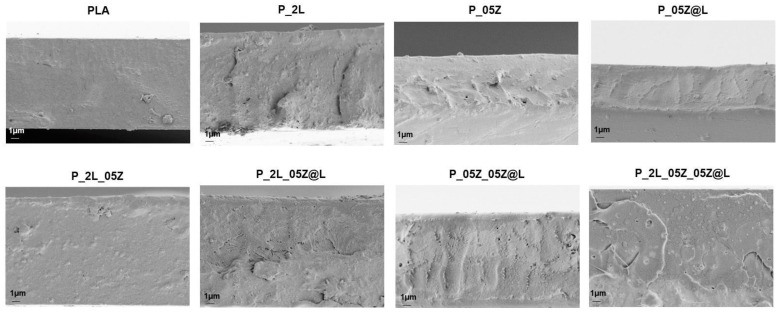
FESEM investigation of fractured surfaces for PLA based formulations.

Figure 4 shows the morphology of fractured surfaces for the different produced PLA nanocomposites. PLA film showed a continuous homogeneous and smooth surface, which was partially preserved in all produced films containing the nanoparticles (LNP, ZnO and ZnO@LNP). However, nanocomposite films were characterized by rougher fractured surface respect to neat PLA, underlining a more brittle tendency of these materials [45,46]. PLA film loaded with 0.5% wt of ZnO shows the presence of nanopores in the internal surface, this characteristic induced not only a different morphological aspect, but could be also responsible for variations in terms of final mechanical performance. The fractured surface of P_05Z@L appeared homogenous and smooth without the presence of any defect; the adopted process guarantees the good dispersion and incorporation of nanofiller (ZnO@LNP) in the polymeric matrix. The morphological differences observed for the different PLA-based films may be associated with the differences in terms of viscosity, already observed during the extrusion process. The formation of nanopores, observed in ternary and quaternary films, can be related to the differences that can arise during the cooling step in the presence of different nanofillers.

**Figure 5:**
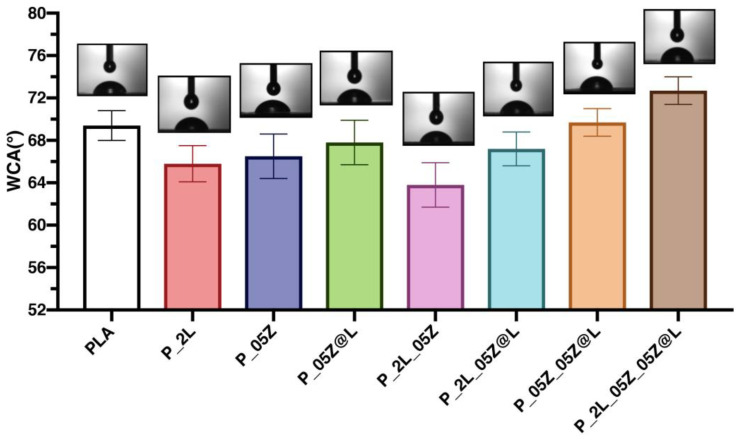
Water contact angles of PLA-based formulations.

Results of water contact angle (WCA) measurements for PLA-based films are reported in Figure 5. We measured a mean value of 70° for neat PLA [44]. The contact angle values, as reported in Figure 5, are quite similar for the various formulations. The contact angle value is directly related to the morphology of the samples, i.e., the surface roughness of the films. The formulations containing the nanoparticles have a greater roughness than that of neat PLA, thus influencing the value of the contact angle. In the present work, the formulations have very similar WCA values, and therefore they are materials with similar behavior in terms of wettability. This characteristic derives from the fact that all the surfaces of the analyzed films are smooth, homogeneous, and without the presence of relevant defects on the external surfaces, as illustrated previously in the morphological analysis at FESEM (Figure 2 and Figure 3). The highest value of the contact angle, a mean value of 73°, was recorded for the quaternary film (P_2L_05Z_05Z@L), and this behavior is induced by the high roughness (see Figure 3 Surfaces-SE2) of the system deriving from the presence of the different nanostructures in the polymer film. These results correlate with that reported in the literature, indicating that the same sample can present different WCA values if the surface is smooth or rough. In fact, roughness induces an increase in WCA values [47].

**Figure 6:**
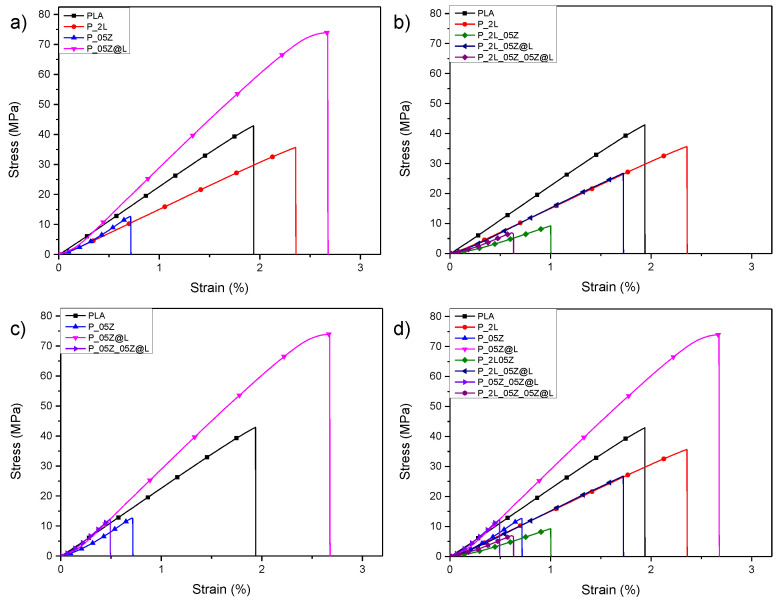
Stress-strain curves of PLA-based formulations. PLA, P-2L, P-05Z and P_05Z@L (**a**). PLA and P2L based nanocomposites (**b**). PLA, P-05Z, P_05Z@L and P-05Z_05Z@L (**c**). Comparison among PLA and PLA-based nanocomposites (**d**).

Figure 6 shows the stress–strain curves of PLA based systems, while Table 2 resumes the characteristic values of the tensile tests. In Figure 6a, the stress–strain curves of PLA and binary based systems (P_2L, P_05Z and P_05Z@L) resulting from tensile tests are reported. The presence of 2% wt. of LNP in PLA matrix induces a reduction of Young’s modulus (E_Young_) and stress at break (σ_b_) in accordance with the literature [46,48,49]. The addition of zinc oxide nanoparticles induces a variation of mechanical performance, the tensile strength and the deformation at break progressively decreased (Table 2 and Figure 6a), highlighting an increase of the brittle nature of PLA matrix [50,51,52]. This behavior is also influenced by the presence of nanopores in the internal surface of P_05Z, as already observed in Figure 4. The presence of hybrid nanoparticles (ZnO@LNP) in PLA matrix improved the mechanical performance. In detail, P_05Z@L film was characterized by higher values of stress at break (σ_b_ = (65.9 ± 9.9) MPa) and Young’s modulus (E_Young_ = (3030 ± 290) MPa) in comparison with neat PLA and all the different produced nanocomposites. However, the deformation at break for P_05Z@L (ε_b_ = (2.4 ± 0.4)%) was maintained unchanged respect to the value registered for PLA (ε_b_ = (2.1 ± 0.1)%) and P_2L (ε_b_ = (2.1 ± 0.6)%). Similar mechanical behavior was observed in literature by Klapiszewski and co-authors who considered hybrid lignin/zinc oxide fillers in polypropylene composites [39]. The presence of hybrid nanoparticles in PLA nanocomposites at 0.5% wt, as reported in this research activity, are able to generate a synergic effect in terms of tensile properties. The positive behavior is associated with the homogeneous distribution of this type of nanofiller in a PLA matrix. Focusing attention on tensile curves of P_2L based nanocomposites (Figure 6b), it is evident that the addition and combination of ZnO and ZnO@LNP nanofillers determine a compromised strength. Comparing the effect induced by the presence and the combination of ZnO and ZnO@LNP nanofillers (Figure 6c) in polymeric systems, it is possible to note that the addition of both nanofillers (P_05Z_05Z@L) determines a reduction of mechanical performance (σ_b_ and ε_b_), while the elastic modulus kept the same trend registered for PLA film (Table 2). The mechanical performance of quaternary film (P_2L_05Z_05Z@L) is wisely reduced, limited by the combination of the different nanoparticles in the polymeric matrix (Figure 6c and Table 2). Finally, Figure 6d shows the stress/strain curves of all produced PLA systems. The higher tensile mechanical properties were observed for P_05Z@L system. Nevertheless, the limited deformation and stress at break values registered for some of the produced formulations did not exclude their use and application in the food-packaging sector: films with low mechanical performance could be used, in the packaging sector, for the realization of internal layers or substrates to be inserted in the packages, while preserving at the same time biocompatibility, that is the basic requirement for final use.

### 3.3. Protein Adsorption

**Figure 7:**
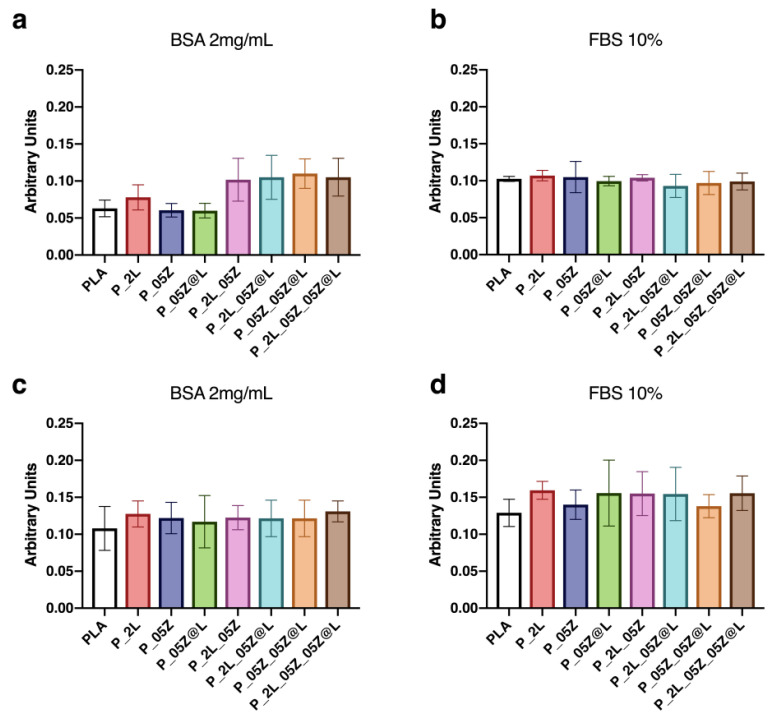
Adsorbed protein on neat PLA, PLA binary, -ternary and -quaternary composite films. (**a**,**b**) Total adsorbed proteins (absorbance 595 nm) from BSA and 10% FBS after 30 min at 37 °C and (**c**,**d**) after 24 h at 37 °C. Data are representative of three independent experiments which yielded similar results.

We evaluated the protein-binding capability of PLA-binary, -ternary and -quaternary nanocomposite films by measuring the protein adsorption values (Figure 7). The assay was conducted according to our protocol, by using 2 mg/mL of the purified protein BSA and 10% FBS [36]. No significant differences were observed in the levels of adsorbed BSA on PLA-binary films after 30 min of incubation compared to PLA film, whereas a slight increase was observed on PLA-ternary and -quaternary films (Figure 7a). After 24 h of incubation, the levels of adsorbed BSA on neat PLA and PLA-binary films increased to the levels of the other films at previous incubation time (Figure 7c). No variations of adsorbed BSA were found on PLA-ternary and -quaternary films, indicating that it reached the saturation levels already at 30 min of incubation (Figure 7c). No significant differences were observed in the levels of adsorbed 10% FBS on PLA-binary, -ternary and -quaternary films after 30 min and 24 h of incubation compared to PLA film (Figure 7b,d), although in the last time point the levels were higher compared to the previous time point (Figure 7d). The absence of differences in the adsorption levels of FBS at 30 min among films may be due to its wide protein composition, by contrast with the unique BSA protein, which might favor binding with the film surface.

### 3.4. Culture of Human Adult Mesenchymal Stem Cells on Neat PLA and PLA-Binary,- Ternary, and -Quaternary Films

**Figure 8:**
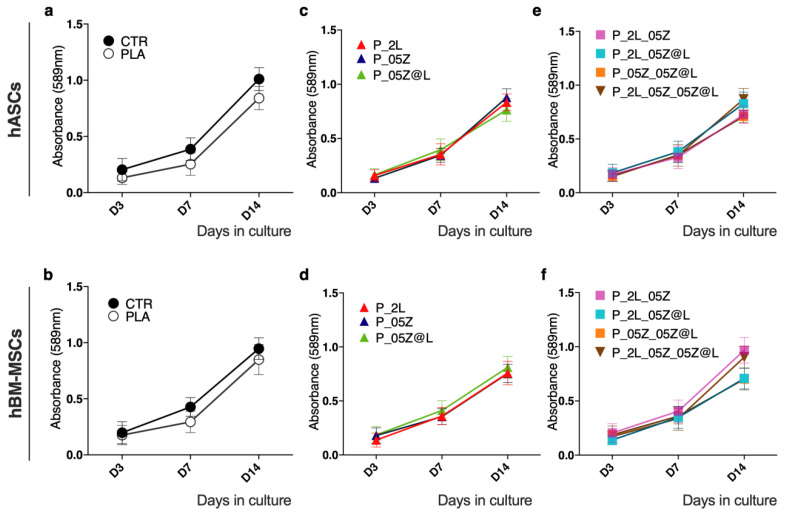
Mesenchymal stem cell viability and proliferation on PLA films. MTT assay of (**a**,**c**,**e**) human adult adipose stem cells (hASCs) and (**b**,**d**,**f**) human bone marrow-mesenchymal stem cells (hBM-MSCs) on PLA, PLA-binary, -ternary, -quaternary films and canonical culture conditions (CTR) at 3, 7 and 14 days. Data are representative of three independent experiments which yielded similar results.

**Figure 9:**
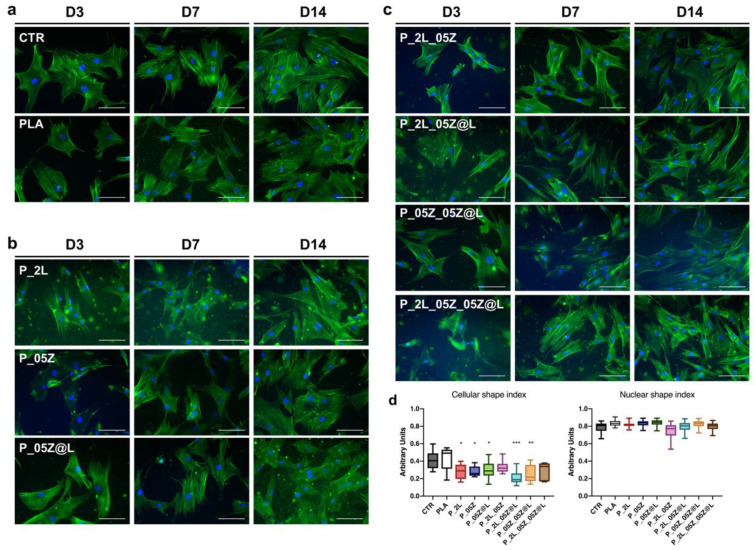
hASCs morphology on PLA films. F-actin fibers of hASCs cultured on (**a**) control and PLA, (**b**) PLA binary (P_2L, P_05Z, P_05Z@L) and (**c**) PLA ternary (P_2L_05Z, P-2L_05Z@L, P05Z_05Z@L) and quaternary (P_2L_05Z_05Z@L) films. F-actin (Alexa-fluor-488 Phalloidin, green), Nuclei (4′,6-diamidino-2-phenylindole, DAPI, blue), Scale bar = 100 m. (**d**) Cell shape index, and nuclear shape index. Cyto-morphometric measurements in Figure 9d refer to stem cells at day 7 in culture, however, these results are representative of hASCs at each time in culture; * *p* < 0.05, ** *p* < 0.01, *** *p* < 0.001.

**Figure 10:**
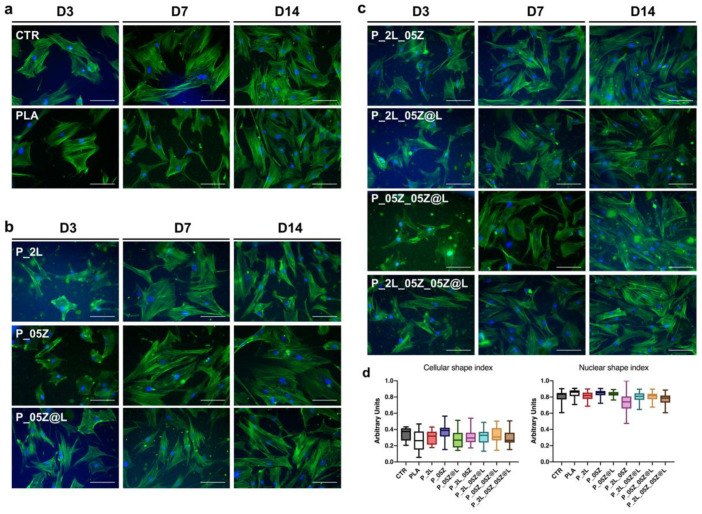
hBM-MSCs morphology on PLA films. F-actin fibers staining of hBM-MSCs cultured on (**a**) CTR and PLA, (**b**) PLA binary (P_2L, P_05Z, P_05Z@L) and (**c**) PLA ternary (P_2L_05Z, P2L_05Z@L, P05Z_05Z@L) and quaternary (P_2L_05Z_05Z@L) films. F-Actin (Alexa-fluor-488 Phalloidin, green), Nuclei (4′,6-diamidino-2-phenylindole, DAPI, blue), Scale bar = 100 m. (**d**) Aspect ratio and nuclear shape index measures at 7 days in culture.

**Figure 11:**
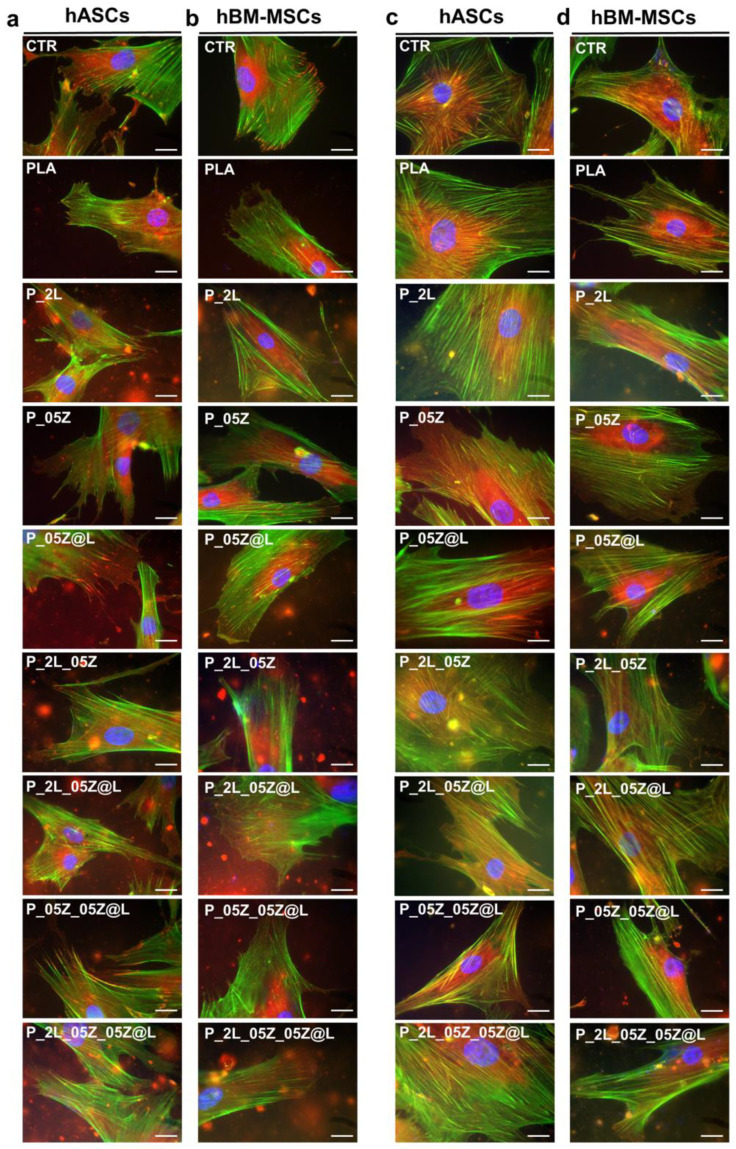
(**a**, **b**) Vinculin expression in hASCs (**a**) and hBM-MSCs (**b**) on PLA and PLA-binary, -ternary and -quaternary films. Representative images of Vinculin spots (anti-Vinculin antibody; RED) in CTR, PLA and PLA-based films. F-Actin (Alexa-fluor-488 Phalloidin, green), Nuclei (4′,6-diamidino-2-phenylindole, DAPI, blue), Scale bar = 20 m. (**c**,**d**) Filamin A expression in hASCs (**c**) and hBM-MSCs (**d**) on PLA and PLA-binary, -ternary and -quaternary films. Representative images of Filamin A (anti-filamin antibody; RED) in CTR, PLA and PLA nanocomposites. F-Actin (Alexa-fluor-488 Phalloidin, green), Nuclei (4′,6-diamidino-2-phenylindole, DAPI, blue), Scale bar = 20 m.

We have evaluated if PLA, PLA-binary, -ternary, and -quaternary nanocomposites were suitable for the culture of human adult adipose stem cells (hASCs) and human adult bone-marrow mesenchymal stem cells (hBM-MSCs). We analyzed the cell viability, proliferation rate, shape and adhesion (Figure 8, Figure 9, Figure 10 and Figure 11). As controls, hBM-MSCs and hASCs were cultured on tissue culture polystyrene (TCP). Cultures were analyzed at different time points (3, 7, 14 days [D]).

#### 3.4.1. Stem Cell Viability and Proliferation on PLA-Binary, -Ternary, and -Quaternary Films

Stem cell viability was monitored by measuring the activity of the mitochondrial dehydrogenase activity with the MTT assay (Figure 8). We observed a comparable enzymatic activity curve in both hASCs and hBM-MSCs on canonical culture conditions (CTR) and neat PLA (Figure 8a,b). No variation of the mitochondrial dehydrogenase curve was observed in hASCs and hBM-MSCs cultured on PLA films (PLA-binary, -ternary and -quaternary films) (Figure 8c–f). Moreover, no signs of toxicity were observed in all cultures. According with the correlation of cell number and MTT absorbance (see Section 2), data reported in Figure 8a,b indicated a comparable proliferation rate of both types of stem cells in control condition and in culture on all PLA films.

These data indicated that the fillers LNP, ZnO and ZnO@LNP on PLA films have no toxic effect on mesenchymal stem cells viability and proliferation rate, therefore neat and PLA nanocomposites films are biocompatible and suitable for mesenchymal stem cell cultures.

#### 3.4.2. Stem Cell Shape on PLA-Binary, -Ternary, and -Quaternary Films

The stem cell morphology was evaluated by the analysis of the cytoskeleton architecture of hASCs and hBM-MSCs seeded on neat and PLA-based films by monitoring the immune-staining of F-actin fibers with Alexa-fluor-488 Phalloidin (Figure 9 and Figure 10).

hASCs cultured on neat PLA adhered to the surface and maintained the canonical mesenchymal fibroblast-like morphology as in control conditions with F-actin stress-fibers crossing the cell cytoplasm as cord stress fibers (Figure 9a, representative images). No cell shape changes were observed overtime in culture (D3, D7, D14, representative images Figure 9a). hASCs adhered on PLA-binary, -ternary and -quaternary film surfaces, but compared to stem cells on PLA and CTR systems that displayed a squared morphology, have a more compact shape as revealed by F-actin stress-fibers that depicted shorter stem cells (Figure 9b,c, representative images).

These findings were confirmed by the morphometric measurements of the cell shape index (CSI) that was significantly lower in PLA-binary, -ternary and -quaternary films compared to PLA value, comparable to CTR (Figure 9d). No significant differences were observed in the nuclear shape index (NSI) values among CTR, PLA and PLA nanocomposite films (Figure 9d).

hBM-MSCs cultured on neat PLA adhered to the surface and maintained the mesenchymal fibroblast-like morphology as in control conditions during the culture time (Figure 10a; representative images). hBM-MSCs adhered to the surface and almost maintained this morphology and behavior also on PLA-binary, -ternary and -quaternary films at each time point evaluated (Figure 10b,c, representative images).

As above, we measured the morphometric descriptors CSI and NSI in hBM-MSCs on PLA and PLA-based films. Data reported in Figure 10d were obtained from stem cells at day 7 in culture, but they were representative of hBM-MSCs at each time point of culture. The CSI values confirmed the absence of significant morphological changes among hBM-MSCs on neat and PLA films and CTR (Figure 10d). Similarly, no NSI differences were found among stem cells on CTR, PLA and PLA-derivative films (Figure 10d).

#### 3.4.3. Interaction of Mesenchymal Stem Cells on Neat PLA and PLA-Binary, -Ternary and -Quaternary Film Surface

To correlate the behavior of hASCs and hBM-MSCs on neat and PLA nanocomposites films we analyzed the interaction of both stem cell types with the surface of each film considered in this study. We analyzed the expression of Vinculin, one of the main components of the focal adhesion complex by which cells establish their interaction with the substrate and transmit the traction force of this binding to the cytoskeleton, which responds by adapting its architecture [26].

Both hASCs and hBM-MSCs established Vinculin focal adhesion spots on all PLA films similarly to CTR culture as displayed in Figure 11a,b, where the connection of Vinculin spots (RED, representative images) with the end of F-actin fibers (GREEN) has a canonical organization in all systems (Figure 11a,b). We observed a widespread distribution of the Vinculin spots in hASCs on PLA-binary, -ternary and -quaternary as on PLA and CTR (Figure 11a). Similarly, no significant differences were observed on the distribution of Vinculin spots in hBM-MSCS on the surface of CTR, neat PLA and PLA-based nanocomposites (Figure 11b).

Finally, we have evaluated the influence of mechanical properties among PLA films in mesenchymal stem cell behavior. We have analyzed the expression of the filamin A (Figure 11c,d, RED), that it is well known to be directly correlated with the mechanical force applied to the cells, including those of a given biomaterial where they are seeded [26,53]. The filamin A is part of the actin-linking protein family, therefore, in the figure is also reported the F-actin staining (GREEN). In Figure 11 is reported the expression of filamin A in hASCs (c) and hBM-MSCs (d) on PLA and PLA-binary, -ternary, and -quaternary films in comparison with the respective control culture. In hASCs, we found a comparable organization of filamin A/F-actin in CTR- and PLA-cultures, while some differences in stem cells on PLA nanocomposites films were observed (Figure 11c). In particular, we detected a distinction of the two protein signals in stem cells on the binary P_05Z, P_05Z@L, ternary P_05Z_05Z@L and quaternary P_2L_05Z_05Z@L films compared to P_2L and ternary P_2L_05Z and P_2L_05Z@L where we observed an overlap of both signals. Interestingly, the overlap was higher in hASCs seeded on nanocomposite films with lower E_Young_ compared to neat PLA (Table 2).

The aforementioned configuration of F-actin-filamin A was also confirmed in hBM-MSCs (Figure 11d). In fact, the comparable organization in CTR- and PLA-cultures and some differences in stem cells on PLA nanocomposites films were observed. However, we observed the distinct fluorescent signals of filamin A and F-actin in stem cells on the binary P_05Z, P_05Z@L, ternary P_05Z_05Z@L and quaternary P_2L_05Z_05Z@L films and their overlapping in stem cell on P_2L and on ternary P_2L_05Z, P_2L_05Z@L. Again, these results were observed in hBM-MSCs seeded on nanocomposite films with lower E_Young_ compared to neat PLA (Table 2). These results suggested that the mechanical properties of the films influenced the organization of F-actin-filamin A in both stem cell types, although only hASCs changed their morphology.

## 4. Discussion

In this work, we have described the design and characterization of a set of novel PLA nanocomposites films with nanoparticles of Lignin, ZnO and the hybrid ZnO@LNP and have investigated their interaction with two types of human adult mesenchymal stem cells: adipose stem cells and bone marrow-mesenchymal stem cells.

Nanoparticles of Lignin, ZnO and the hybrid ZnO@LNP were combined in a different formulation generating seven PLA nanocomposites, that were grouped as PLA(P)-binary (P_2L; P_05Z; P_05Z@L), PLA-ternary (P_2L_05Z; P_2L _05Z@L; P_05Z _05Z@L) and PLA-quaternary (P_2L_05Z_05Z@L) films. These films were developed in order to preserve food quality and avoid cell cytotoxicity with the aim of generating suitable films for food industrial and biomedical applications. In particular, considering the presence of naturally antioxidant and UV-blocking lignin that has been shown to have magnified properties at the nanoscale [53], the PLA-based films presented in this work could be used in active food packaging, of great significance in the food sector, as they could favor the reduction of both oxidation and deterioration of physical and organoleptic properties (such as color and flavor). Additionally, the physicochemical properties of both neat and nanocomposites PLA, such as their low thickness and mechanical properties, suggest that they could be used for the development of biohybrid systems for tissue-engineering purposes.

In this regard, we performed a thorough characterization of PLA and PLA-binary -ternary and -quaternary films. Comparable values were found for water wettability and similar surface morphologies were observed for all the formulations, while a noticeable difference was measured in the mechanical (tensile) performance of the different hybrid films. In fact, while the presence of LNP and ZnO, both in PLA binary and ternary systems, induced an overall reduction of elastic modulus, stress and deformation at break, in the case of hybrid systems the inclusion of ZnO@LNP in the PLA films generated a visible increase in the stiffness and tensile strength.

Of note, all neat and nanocomposites PLA films were suitable for the culture of adult hASCs and hBM-MSCs. Both stem cell types maintained the same proliferation rate and did not show signs of toxicity on all PLA films as in control culture conditions. Moreover, both stem cell type adhered to the surface of all PLA films, establishing canonical Vinculin adhesion spots as in control systems. Vinculin spots are the key cell components of the focal adhesion (FA) complexes by which they interact with the surface [26,54,55,56]. In particular the FA complex, through the transmembrane protein integrins, establishes a connection between the F-actin fibers and the extracellular matrix and is directly involved in the maintenance of the cell homeostasis. Therefore, the presence of Vinculin spots in both stem cell-PLA systems confirmed the absence of adverse effects of the films’ characteristics on stem cells. We found a wide distribution of Vinculin spots in hASCs and in hBM-MSCs on nanocomposites PLA as on neat PLA and control system. These findings correlated with the data of protein adsorption on all films that showed a strong interaction of proteins with the films’ surface. Interestingly, we measured a different CSI in hASCs seeded on nanocomposites films compared to neat PLA and control system, whereas no differences were observed in hBM-MSCs on all PLA-derived films and control. We interpreted these results as a specific different response of the two types of stem cell to the surfaces of PLA-based films. It is well demonstrated that stem cells interact with the surface of films or scaffolds recapitulating their physiological contact with the extracellular matrix [57,58,59], thus different types of stem cell might respond differently to the same film surface or to the same biomaterial property (e.g., elasticity, nanotopography) [60,61,62,63]. Therefore, we were not surprised to observe the different responses of hASCs and hBM-MSCs on PLA film surfaces.

Both stem cells respond to the different films’ mechanical properties orchestrating the organization of Filamin A towards the F-actin fibers on neat PLA and PLA nanocomposite films. Filamin A is an actin-binding protein involved in the structural organization of the cytoskeletal F-actin and in the connections of the F-actin fibers with the FA complex and by these to transmembrane receptors and extracellular matrix [64]. More importantly, filamin A, through cross-linking, senses the mechanical properties of the extracellular matrix and responds reorganizing the F-actin cytoskeleton [26,54,64]. In accordance with the different mechanical characteristics of PLA films, we found a different organization of filamin A towards F-actin in both stem cell types on the binary P_05Z, P_05Z@L, ternary P_05Z_05Z@L and quaternary P_2L_05Z_05Z@L films compared to the binary P_2L and the ternary P_2L_05Z and P_2L_05Z@L with respect to PLA and control. In particular, we observed the higher correspondence of the fluorescent signal of two proteins on nanocomposite films where mechanical properties (E_Young_) were lower, indicating that cells respond actively to the mechanical cues of the films. Interestingly, these films have also the higher wettability compared to neat PLA and the other nanocomposites. This feature may explain the absence of overlap of both protein signal in the quaternary system were the E_Young_ and the wettability were both lower compared to PLA.

Collectively, these results demonstrated that neat and nanocomposites PLA-derived films containing LNP, ZnO and ZnO@LNP were suitable for the cultures of stem cells and for developing biomedical applications. Moreover, the biocompatibility with human stem cells also represents a proof of the safety of neat and nanocomposites PLA films for food application purposes.

## Figures and Tables

**Table 1:** Material formulations.

Formulations	Acronyms	PLA (wt.%)	LNP (wt.%)	ZnO (wt.%)	ZnO@LNP (wt.%)
PLA	PLA	100		-	-
PLA_2LNP	P_2L	98	2	-	-
PLA_0.5ZnO	P_05Z	99.5	-	0.5	-
PLA_0.5ZnO@LNP	P_05Z@L	99.5	-	-	0.5
PLA_2LNP_0.5ZnO	P_2L_05Z	97.5	2	0.5	-
PLA_2LNP_0.5ZnO@LNP	P_2L_05Z@L	97.5	2	-	0.5
PLA_0.5ZnO_0.5ZnO@LNP	P_05Z_05Z@L	99	-	0.5	0.5
PLA_2LNP_0.5ZnO_0.5ZnO@LNP	P_2L_05Z_05Z@L	97	2	0.5	0.5

PLA = polylactic acid; LNP = lignin nanoparticles; ZnO = Zinc Oxide nanoparticles; ZnO@LNP = ZnO on LNP nanoparticles.

**Table 2:** Mechanical properties of PLA-based systems.

Formulations	σ_b_ (MPa)	ε_b_ (%)	E_Young_ (MPa)
PLA	37.4 ± 7.4	2.1 ± 0.1	2280 ± 210
P_2L	20.8 ± 9.2	2.1 ± 0.6	1280 ± 210
P_05Z	11.8 ± 1.5	0.7 ± 0.1	2120 ± 230
P_05Z@L	65.9 ± 9.9	2.4 ± 0.4	3030 ± 290
P_2L_05Z	8.6 ± 3.2	0.9 ± 0.1	1115 ± 200
P_2L_05Z@L	26.6 ± 4.7	2.0 ± 0.4	1490 ± 175
P_05Z_05Z@L	13.9 ± 5.3	0.8 ± 0.3	2315 ± 420
P_2L05Z_05Z@L	6.6 ± 2	0.6 ± 0.2	1267 ± 86

σ_b_: Stress at break; ε_b_: deformation at break; E_Young_: Young’s modulus.
